# Clinical Characteristics and Outcomes of SARS-CoV-2 Infection in Neonates with Persistent Pulmonary Hypertension of the Newborn (PPHN): A Systematic Review

**DOI:** 10.3390/children11111305

**Published:** 2024-10-28

**Authors:** Saad Alhumaid, Muneera Alabdulqader, Zainab Al Alawi, Mohammed A. Al Ghamdi, Mohammed A Alabdulmuhsin, Hassan I Al Hassar, Hussain Ahmed Alsouaib, Hussain Ali Alhassan, Hassan Al-Helal, Sameer Ahmed Almoraihel, Mohammed Jaber Alomran, Hassan Redha AL-Tarfi, Abbas Radi Al-Makinah, Tariq T. Alghareeb, Mohammad Abdullah Alkhwaitem, Murtadha Alsuliman, Ali N. Bukhamseen, Khulood Khaled Alajmi, Ahmed Salman Al Majhad, Mariam Ali Almajhad, Ayat Hussain Alhmed, Abdulrahman A. Alnaim

**Affiliations:** 1 School of Pharmacy, University of Tasmania, Hobart 7000, Australia; 2 Pediatric Nephrology Specialty, Pediatric Department, Medical College, King Faisal University, Al-Ahsa 31982, Saudi Arabia; malabdulqader@kfu.edu.sa; 3 Division of Allergy and Immunology, College of Medicine, King Faisal University, Al-Ahsa 31982, Saudi Arabia; zalalwi@kfu.edu.sa; 4 Department of Pediatrics, King Fahad Hospital of the University, College of Medicine, Imam Abdulrahman Bin Faisal University, Dammam 34212, Saudi Arabia; moalghemdi@iau.edu.sa; 5 Pharmacy Department, Maternity and Children Hospital, Ministry of Health, Al-Ahsa 36422, Saudi Arabia; moalabdulmohsin@moh.gov.sa (M.A.A.); hualhasan@moh.gov.sa (H.A.A.); abokhamsin@moh.gov.sa (A.N.B.); 6 Pharmacy Department, Aljafr General Hospital, Ministry of Health, Al-Ahsa 7110, Saudi Arabia; hhassar@moh.gov.sa (H.I.A.H.); sal-moraihel@moh.gov.sa (S.A.A.); 7 Medical Store Department, Maternity and Children Hospital, Ministry of Health, Al-Ahsa 36422, Saudi Arabia; halsouaib@moh.gov.sa (H.A.A.); haltarfi@moh.gov.sa (H.R.A.-T.); asalmajhad@moh.gov.sa (A.S.A.M.); 8 Division of Laboratory, Medical Microbiology Department, Maternity and Children Hospital, Ministry of Health, Al-Ahsa 36422, Saudi Arabia; haalalhelal@moh.gov.sa; 9 Medical Department, Aljafr General Hospital, Ministry of Health, Al-Ahsa 7110, Saudi Arabia; mojalomran@moh.gov.sa; 10 Dental Department, Aljafr General Hospital, Ministry of Health, Al-Ahsa 7110, Saudi Arabia; aralmakina@moh.gov.sa; 11 Administration of Pharmaceutical Care, Al-Ahsa Health Cluster, Ministry of Health, Al-Ahsa 36421, Saudi Arabia; talghareeb@moh.gov.sa; 12 Pharmacy Department, Rumah General Hospital, Ministry of Health, Riyadh 11981, Saudi Arabia; malkhowaitem@moh.gov.sa; 13 Department of Pharmacy, Hereditary Blood Diseases Centre, Ministry of Health, Al-Ahsa 36422, Saudi Arabia; moalsalman@moh.gov.sa; 14 Pharmacy Department, Al-Ahsa Mental Health Hospital, Ministry of Health, Al-Ahsa 31982, Saudi Arabia; kalajmi@moh.gov.sa; 15 Nursing Department, Maternity and Children Hospital, Ministry of Health, Al-Ahsa 36422, Saudi Arabia; maalmajhad@moh.gov.sa; 16 Administration of Nursing Care, Maternity and Children Hospital, Ministry of Health, Al-Ahsa 36422, Saudi Arabia; ayatha@moh.gov.sa; 17 Department of Pediatrics, College of Medicine, King Faisal University, Al-Ahsa 31982, Saudi Arabia; ahalnaim@kfu.edu.sa

**Keywords:** COVID-19, persistent fetal circulation, patent ductus arteriosus, patent foramen ovale, persistent pulmonary hypertension, PPHN, risk, SARS-CoV-2, severity

## Abstract

PPHN is a common cause of neonatal respiratory failure and is still a serious condition that is associated with high mortality. Objectives: To analyze the clinical characteristics and outcomes of SARS-CoV-2 infection in neonates with PPHN to identify neonatal cases at risk to develop severe illness. Methods: For this systematic review, we adhered to the Preferred Reporting Items for Systematic Reviews and Meta-Analyses (PRISMA) guidelines and searched Medline, Embase, CINAHL, and PubMed for studies on the development of COVID-19 in neonates with PPHN, published from 1 December 2019 to 29 February 2024, with an English language restriction. Results: Of the 2406 papers that were identified, 21 articles were included in the systematic review. Studies involving thirty-six neonates with PPHN and infected with SARS-CoV-2 were analyzed (twenty-nine survived, six died, and one is still hospitalized). The main causes of PPHN in neonates who had COVID-19 were neonatal respiratory distress syndrome (NRDS) (41.7%), meconium-stained amniotic fluid (MSAF) (16.7%), preterm premature rupture of membranes (PPROM) (11.1%), hypoxic ischemic encephalopathy (HIE) (5.5%), pneumonia (5.5%), and idiopathic (2.8%). Most of those neonates were male (33.3%), belonged to Indian ethnicity (50%), and were delivered via caesarean section (44.4%). COVID-19 in cases with PPHN commonly occurred in neonates born with a pregnancy range from 32 to <37 weeks (moderate to late preterm) (36.1%). The maternal severity of COVID-19 was reported to be severe in three cases only (8.3%); however, SARS-CoV-2 infection in neonates with PPHN was either severe (44.4%) or critical (22.2%). Most of these neonates experienced acute respiratory distress syndrome (ARDS) (58.3%). Early and late multisystem inflammatory syndrome in neonates (MIS-N) were reported in 50% and 11.1%, respectively. A high proportion of neonates were admitted to the intensive care unit (ICU) (58.3%) or needed mechanical ventilation (MV) (47.2%). Neonates with concurrent PPHN and SARS-CoV-2 infection who died had worse severity of COVID-19 [i.e., severity of COVID-19 was critical in 10% (neonates with PPHN who survived group) vs. 83.3% (neonates with PPHN who died group); *p* = 0.026]. Neonates with PPHN and COVID-19 had a higher relative risk of death if they received more antibiotics (RR 4.14, 95% CI 0.64–6.88) and if their COVID-19 was defined as critical (RR 2.84, 95% CI 0.86–9.39). Male neonates with PPHN and COVID-19 (RR 2.60, 95% CI 0.30–1.17) and those requiring prolonged invasive positive pressure ventilation (RR 2.22, 95% CI 0.64–7.73) also showed an increased relative risk for death. Conclusions: COVID-19 in neonates with PPHN is challenging and may be associated with increased mortality, severity, ICU admission, ARDS, MIS-N, and MV usage. The results should be interpreted with caution owing to the small number of studies and substantial heterogeneity and indicate a need for future research in this area. Due to its benefits, testing for SARS-CoV-2 should be encouraged for newborns with symptoms consistent with COVID-19, especially in neonates with a history of SARS-CoV-2 exposure. Effective protection measures should be implemented during delivery and post-delivery care as necessary.

## 1. Introduction

Despite advances in neonatal cardiorespiratory care, persistent pulmonary hypertension of the newborn (PPHN) remains a serious condition and is associated with a high mortality rate, ranging between 7% and 35.7% [1,2,3,4]. The most commonly involved systems in PPHN are cardiovascular and respiratory systems with echocardiographic abnormalities and raised cardiac biomarkers; therefore, neonates with PPHN of all ages are assumed to be at high risk for adverse health outcomes from coronavirus disease 2019 (COVID-19). PPHN can cause acute respiratory failure and in many COVID-19 cases, patients have been reported to experience acute respiratory distress syndrome (ARDS) [5]; thus, the co-existence of both conditions can be deleterious. COVID-19 can impact pulmonary blood flow through various mechanisms, some of which overlap with the disease process of PPHN. These mechanisms include the following: (1) inflammation, swelling, and damage of the endothelial cells and blood vessels; (2) small blood vessel clots; (3) blood clots in the veins; and (4) changes in blood flow dynamics due to ARDS and the use of mechanical ventilation.

It is uncertain which PPHN neonates are at high risk for a worse COVID-19 clinical course, although there is an increasing number of studies reporting on real-world patients who suffered PPHN and COVID-19 concurrently [6,7,8]. Recent data indicate that neonatal cases with PPHN may not be at greater risk than others [8,9]; however, some early studies in neonates with PPHN suggested that the risk of severe COVID-19 is higher in this population [10,11,12]. There is limited evidence on the final treatment outcome in neonates who had PPHN and were infected with severe acute respiratory syndrome coronavirus 2 (SARS-CoV-2), as findings were built on very few cases or small sample populations and many of these studies reported conflicting results [13,14,15]. Due to the lack of comprehensive and updated systematic reviews focusing on COVID-19 in neonates with PPHN, we aimed to analyze the clinical characteristics and outcomes of SARS-CoV-2 infection in neonates with PPHN to identify neonatal cases at risk to develop severe illness, to decrease the mortality rate among those patients, and improve their final healthcare outcomes.

## 2. Methods

### 2.1. Design

We followed the recommendations of the Preferred Reporting Items for Systematic reviews and Meta-Analysis (PRISMA) statement to perform this systematic review [16]. The systematic review protocol was entered into the International Prospective Register of Systematic Reviews (PROSPERO) under CRD42024596354. Published articles from 1 December 2019 to 29 February 2024, with an English language restriction, were selected for review from four electronic databases (Medline, Embase, CINAHL, and PubMed). The search phrases included Boolean terms ‘AND’ and ‘OR’ with the following keywords in various possible combinations: “pulmonary hypertension”, “patent ductus arteriosus”, “patent foramen ovale”, “PPHN”, “neonate”, “neonates”, “newborn”, “newborns”, “neonatal”, “COVID-19”, “SARS-CoV-2”, “severe acute respiratory syndrome coronavirus 2”, “coronavirus disease 2019”, and “2019 novel coronavirus” (see Supplementary Table S1 for database-specific search syntax). Articles discussing and reporting the development of COVID-19 in neonates with PPHN were selected based on the title and abstract.

The PRISMA 2020 checklist was used (see Supplementary Materials) [16].

### 2.2. Inclusion–Exclusion Criteria

We included observational studies [case reports, case-series and cohort studies] that reported the real-world development of COVID-19 in neonates with PPHN. We excluded editorials, commentaries, expert opinions, discussion papers, reviews, meta-analyses, or studies available in languages other than English.

### 2.3. Definitions of COVID-19 Severity, Mother-to-Neonate Transmission of SARS-CoV-2, and Multisystem Inflammatory Syndrome in Neonates (MIS-N)

The severity of COVID-19 was defined as per the Clinical Spectrum of SARS-CoV-2 Infection issued by the National Institutes of Health (i.e., asymptomatic, mild, moderate, severe, and critical) [17].

We applied the World Health Organization (WHO) definition to assess for the mother-to-neonate transmission in neonates with a positive SARS-CoV-2 reverse transcriptase polymerase chain reaction (RT-PCR) or immunoglobulin M (IgM) test [18]. Cases were categorized as confirmed/possible/unlikely/indeterminate for intrauterine, intra- partum, or early postpartum infection. Stillborn babies were not included in our analysis [18].

MIS-N was defined according to the current United States Centers for Disease Control and Prevention case definition in an individual aged < 28 days [19,20]. The neonates who fulfilled all the criteria for the definition were termed as “most likely MIS-N”. The neonates who presented with a high suspicion for MIS-N but could not fulfil all the criteria were termed as “possible MIS-N” if no alternative diagnosis was suggested, or “unlikely MIS-N” if an alternative diagnosis was available. The neonates who presented within the first 3 days of life were termed as early, and those who presented beyond 3 days until 28 days of life were termed as late MIS-N [21].

### 2.4. Data Extraction

Six reviewers—Saad Alhumaid, Muneera Alabdulqader, Zainab Al Alawi, Mohammed A Alabdulmuhsin, Hassan I Al Hassar, and Hussain Ahmed Alsouaib—independently screened the papers by reviewing titles and abstracts based on the selection criteria. If there was disagreement and agreement could not be reached after discussion between the two investigators, a third investigator adjudicated.

A standardized data collection form was used to collate information from the studies that we selected and facilitate study quality assessment and data analysis (see Supplementary Tables S2 and S3 for summary of the characteristics of the included studies, main outcome measures, and clinical features in neonates with PPHN and COVID-19 (*n* = 21 studies), 2020–2023).

### 2.5. Quality Assessment

Two tools were used appropriately to assess the quality of the studies included in this review: (1) Modified Newcastle–Ottawa Scale (NOS) to evaluate case report and case-series studies [22]; and (2) NOS to evaluate cohort studies [23]. The quality assessment was carried out by six co-authors—Hussain Ali Alhassan, Hassan Al-Helal, Sameer Ahmed Almoraihel, Mohammed Jaber Alomran, Hassan Redha AL-Tarfi, and Abbas Radi Al-Makinah—who independently evaluated potential bias using these two tools.

### 2.6. Data Analysis

Descriptive statistics were used to describe the data. For categorical variables, frequencies and percentages were reported. Differences between the COVID-19-infected PPHN cases who survived and COVID-19-infected PPHN cases who died were analyzed using the Chi-square (*χ*^2^) tests (or Fisher’s exact tests for expected cell count <5 in more than 20% of the cells). Relative risks (RRs) and 95% confidence intervals (CIs) of the association of each demographic parameter and clinical variable with the treatment outcomes (i.e., survived or died) of PPHN cases with SARS-CoV-2 infection were calculated. All *p*-values were based on two-sided tests and significance was set at a *p*-value less than 0.05. Microsoft Excel 2019 (Microsoft Corp., Redmond, WA, USA) and IBM SPSS Statistics software, version 26.0 (IBM Corp., Armonk, NY, USA) were used for all data wrangling and statistical analyses. The graphical abstract was created with BioRender.com (accessed on 20 October 2024) (agreement no. VG27G3WWB6).

## 3. Results

### 3.1. Study Characteristics and Quality

**Figure 1:**
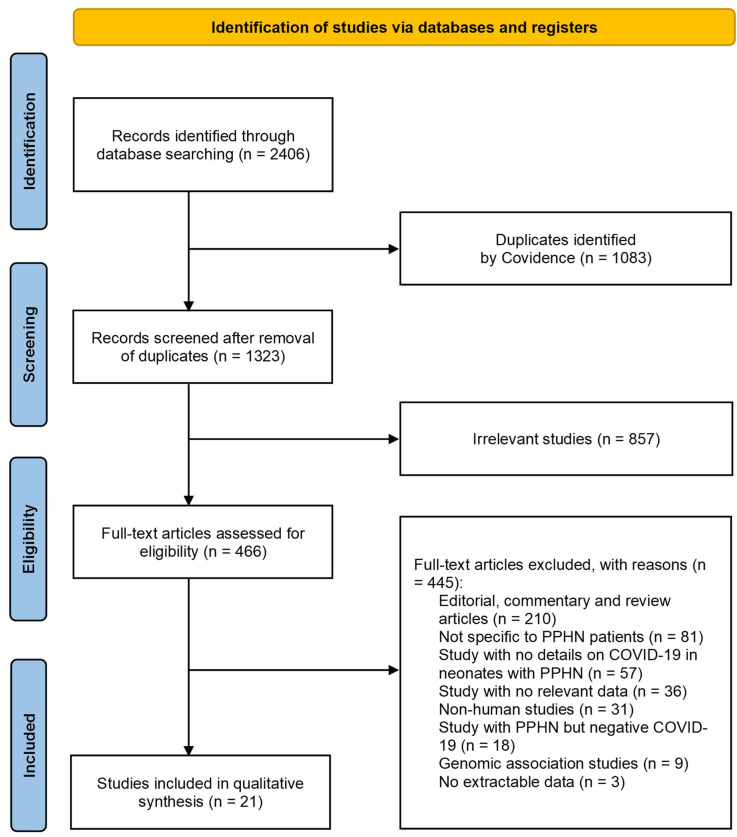
Flow diagram of studies included in the systematic review.

A total of 2406 publications were identified (Figure 1). After the exclusion of duplicates and articles that did not fulfil the study inclusion criteria, twenty-one articles were included in the qualitative synthesis [5,6,7,8,9,10,11,12,13,14,15,24,25,26,27,28,29,30,31,32,33]. The detailed characteristics of the included studies for neonates who were infected with SARS-CoV-2 and had PPHN are shown in Supplementary Tables S2 and S3. There were fourteen case reports [5,9,10,11,12,14,24,25,27,28,29,30,32,33], six case-series studies [6,7,13,15,26,31], and one cohort study [8]. All studies included in this review were retrospective in design except for two studies, which were prospective (*n* = 2) [6,8]. These studies were conducted in India (*n* = 7) [6,8,13,14,15,24,31], United States (*n* = 4) [5,25,26,29], Saudi Arabia (*n* = 2) [10,33], Iran (*n* = 2) [9,27], Turkey (*n* = 1) [11], Brazil (*n* = 1) [12], Israel (*n* = 1) [7], Bangladesh (*n* = 1) [28], the Netherlands (*n* = 1) [32], and Canada (*n* = 1) [30]. All were single-center studies, except for three multi-center studies [6,13,15]. All case reports and case-series studies were assessed for bias using the modified NOS. Thirteen studies were deemed to have high methodological quality [9,10,11,12,15,24,27,28,29,30,31,32,33] and seven were deemed to have moderate methodological quality [5,6,7,13,14,25,26]. The only study that utilized a cohort design had a moderate risk of bias based on NOS [8].

### 3.2. Demographic Features and Clinical Characteristics of SARS-CoV-2 Infection in Neonates with PPHN

The included studies had a total of 36 neonatal cases with PPHN who were infected with SARS-CoV-2, as detailed in Supplementary Tables S2 and S3. The median interquartile range (IQR) age of hospitalized neonates was 90 min (1 to 1440), with an increased male predominance in neonates diagnosed with PPHN and COVID-19 in most of the studies (12/36 = 33.3%) [5,11,12,14,15,24,26,27,29,30,31]. A majority of the neonates belonged to Indian ethnicity (*n* = 18/36, 50%) [6,8,13,14,15,24,31], and seven studies reported the following neonatal ethnicities: Persian (*n* = 2) [9,27], White (Caucasian) (*n* = 1) [11], Black (*n* = 1) [5], Hispanic (*n* = 1) [12], Asian (*n* = 1) [33], and Arab (*n* = 1) [10]. Only two neonates were born via the normal spontaneous vaginal delivery (NSVD) (*n* = 2/36, 5.5%) [27,29]; however, a higher proportion of these neonates were born via the caesarean delivery mode (C-section) (*n* = 16/36, 44.4%) [5,6,9,10,11,12,24,25,28,30,32]. One neonate was born by induction of labor [33]. Around ten (*n* = 10/36, 27.8%) of the neonates were born with a normal birthweight (≥2500 g) [6,15,24,26,27,30,33], but a high proportion of neonates were delivered with low birthweight (≥1500 g to <2500 g) (*n* = 9/36, 25%) [5,6,9,10,11,12,28,31,32]. Two cases were born with extremely low birthweight (<1000 g) [6,25] and one neonate was born with a very low birthweight (<1500 g) [6]. Some neonates were born with a normal gestational age (term: ≥37 weeks) (*n* = 9/36, 25%) [6,9,14,24,26,27], but most neonates were born with a pregnancy range from 32 to <37 weeks (moderate to late preterm) (*n* = 13/36, 36.1%) [5,6,10,11,12,15,28,29,30,31,32,33]. Three cases were very preterm (28 to <32 weeks) (*n* = 3/36, 8.3%) [6,25]. The Apgar score for these neonates was low (range of 0 to 3) (*n* = 7/36, 19.4%) [5,6,25,30,32], moderately abnormal (range of 4 to 6) (*n* = 11/36, 30.5%) [5,6,11,27,30,32], and reassuring (range of 7 to 10) (*n* = 17/36, 47.2%) [6,9,10,11,12,24,27,28,33].

The most frequent medical comorbidities in neonates with PPHN who had moth-er-to-neonate SARS-CoV-2 transmission were arrhythmia (*n* = 6) [6], atrial thrombi (*n* = 6) [6], seizures (*n* = 5) [12,13], hypotension (*n* = 4) [5,11,15,25], intracranial hemorrhage (ICH) (*n* = 4) [5,10,25,32], necrotizing enterocolitis (NEC) (*n* = 3) [6,9,24], sepsis (*n* = 3) [5,11,12], metabolic acidosis (*n* = 3) [10,29,33], decreased fetal movements (*n* = 3) [5,6], lack of fetal movements (*n* = 3) [6,32], chorioamnionitis (*n* = 3) [5,12,25], food intolerance (*n* = 3) [9,24,31], respiratory acidosis (*n* = 2) [5,27], rash (*n* = 2) [9,24], decreased fetal heart rate (*n* = 2) [9,24], vomiting (*n* = 2) [9,24], catecholamine-resistant vasodilatory shock (CRVS) (*n* = 2) [6], shock (*n* = 2) [15], cardiorespiratory failure (*n* = 2) [10,12], hydrocephalus (*n* = 2) [5,9], and hypoxic ischemic encephalopathy (HIE) (*n* = 2) [26,30]. The causes of PPHN were neonatal respiratory distress syndrome (NRDS) (*n* = 15/36, 41.7%) [5,6,9,10,11,15,25,26,28,29,30,32,33]; meconium-stained amniotic fluid (MSAF) (*n* = 6/36, 16.7%) [5,6,25,27]; preterm premature rupture of membranes (PPROM) (*n* = 4/36, 11.1%) [5,6,12,27]; hypoxic ischemic encephalopathy (HIE) (*n* = 2/36, 5.5%) [26,30]; pneumonia (*n* = 2/36, 5.5%); and idiopathic (*n* = 1/36, 2.8%) [24]. The individual oxygen index (OI) for PPHN patients infected with COVID-19 was reported by two studies only and the patient’s OI was moderate (≥15 and <25) (1/36, 2.8%) [10] or severe (≥25 and <40) (1/36, 2.8%) [11]. One study reported on the mean OI for the entire study population (*n* = 10) [26]. As expected, most common therapies initiated to treat PPHN were invasive positive pressure ventilation (IPPV) (*n* = 18/36, 50%) [5,6,7,10,13,14,15,24,27,29,32,33], continuous positive airway pressure (CPAP) (*n* = 17/36, 47.2%) [5,6,7,9,10,11,12,13,24,27,28,29,30,33], inhaled nitric oxide (iNO) (*n* = 14/36, 38.9%) [5,6,7,10,11,14,25,26,29,30,32,33], high frequency ventilation (HFV) (*n* = 10/36, 27.8%) [5,6,10,11,13,24,25,33], surfactant (*n* = 9/36, 25%) [5,9,10,11,12,25,27,29,32], sildenafil (*n* = 9/36, 25%) [9,11,12,14,15,24,27,28,30], noninvasive positive pressure ventilation (NPPV) (*n* = 8/36, 22.2%) [12,13,27,28,31], dopamine (*n* = 7/36, 19.4%) [5,6,9,10,11,24,27], hydrocortisone (*n* = 7/36, 19.4%) [5,6,10,12,25,32,33], packed red blood cells (*n* = 6/36, 16.7%) [5,9,10,25,28,29], milrinone (*n* = 5/36, 13.9%) [6,9,15,27,30], inotropes (*n* = 5/36, 13.9%) [9,14,26,31,32], fresh frozen plasma (*n* = 4/36, 11.1%) [9,25,28,33], diuretics (*n* = 4/36, 11.1%) [9,14,24,28], epinephrine (*n* = 4/36, 11.1%) [6,10,15,25], dobutamine (*n* = 4/36, 11.1) [10,11,27,33], antihypertensives (*n* = 4/36, 11.1%) [9,12,14,27], magnesium sulfate (*n* = 3/36, 8.3%) [6,25,27], vasopressors (*n* = 2/36, 5.5%) [12,14], sedation (*n* = 2/36, 5.5%) [5,30], cardiopulmonary resuscitation (*n* = 2/36, 5.5%) [5,10], platelets (*n* = 2/36, 5.5%) [10,29], endothelin receptor antagonists (ERAs) (*n* = 2/36, 5.5%) [12,14], opioids (*n* = 2/36, 5.5%) [5,27], vasodilators (*n* = 2/36, 5.5%) [14,28], and therapeutic hypothermia (*n* = 2/36, 5.5%) [26,27]. Only one patient was placed on extracorporeal membrane oxygenation (ECMO) with the initial ECMO venoarterial mode [26].

### 3.3. Demographic Features and Clinical Characteristics of SARS-CoV-2 Infection in Mothers Who Delivered Neonates with PPHN and a History of Mother-to-Neonate SARS-CoV-2 Transmission

The most common medical comorbidities in pregnant mothers who had SARS-CoV-2 infection and delivered neonates with PPHN were gestational diabetes mellitus (*n* = 4) [5,10,24,28], pregnancy-induced hypertension (*n* = 3) [10,28,29], preeclampsia (*n* = 3) [25,29,33], coinfections (*n* = 3) [including Group B *Streptococcus* (*n* = 1), *Cytomegalovirus* (*n* = 1) and *Parvovirus B19* (*n* = 1)] [5,32], hypothyroidism (*n* = 2) [27,28], and placenta previa (*n* = 2) [6,25]. However, three mother had no medical comorbidities and were previously healthy (3/36, 8.3%) [9,12,30]. Only three studies reported on the vaccination status against SARS-CoV-2 for mothers who delivered PPHN patients infected with COVID-19, and none of these mothers were vaccinated against SARS-CoV-2 before or during pregnancy or at the time of delivery [5,6,31]. Studies reported a different maternal onset of symptoms for SARS-CoV-2 infection: 3 days or 5 days before hospital admission [12,28,32]; 1 day after hospital admission [29]; 1 day, 12 days, 19 days, or 21 days before birth [9,10,24,33]; and 1 day after birth [10]. Only a few studies reported maternal SARS-CoV-2 infection in pregnant women with a history of close contact with a COVID-19 patient (three cases) [6,31,32] or who had abnormal chest x-rays (including patchy consolidation, or multifocal pneumonia and airspace opacities) (three cases) [10,25,28].

Maternal RT-PCR tests for SARS-CoV-2 were positive in eighteen mothers (18/36, 50%) [5,6,7,9,10,11,12,15,25,27,28,29,30,32,33]. Of the total number of mothers who had positive serologic SARS-CoV-2 antibodies (immunoglobulin G only) perinatally from blood specimens (6/36, 16.7%) [6,24,31,32], all were tested by RT-PCR-SARS-CoV-2 except one [31], and only one mother tested positive for SARS-CoV-2 [32]. The diagnosis of maternal SARS-CoV-2 infection was made by RT-PCR testing of the nasopharyngeal swab samples (*n* = 9) [9,10,11,12,25,28,29,32,33], oropharyngeal swab samples (*n* = 2) [28,32], vaginal secretion sample (*n* = 1) [32], blood sample (*n* = 1) [32], and urine sample (*n* = 1) [32]. Only one study demonstrated the presence of SARS-CoV-2 in the cells of placenta by utilizing an immunohistochemical investigation for SARS-CoV-2 antigen expression in combination with SARS-CoV-2 RNA in situ hybridization [32]. Maternal SARS-CoV-2 positivity was confirmed in another 9 cases using RT-PCR, but the location of samples was not stated [5,6,7,15,27]. One study reported a positive SARS-CoV-2 test in a mother at hospital admission but the method of COVID-19 testing used and location of sample were not mentioned [30]. Two studies used whole-genome sequencing of SARS-CoV-2 and identified similar variants in mothers and their neonates (Gamma and Delta variants) [12,30].

Some mothers tested positive for COVID-19 within 1 day at the hospital admission (*n* = 6) [5,12,28,29,30,32]. However, few mothers tested positive for SARS-CoV-2 after birth by 2 days (*n* = 2) [9,32], 3 days (*n* = 3) [10,27,32], 5 days (*n* = 1) [32], 10 days (*n* = 1) [33], or 19 days (*n* = 1) [24]. Overall, COVID-19 in mothers resulted in no or low severity of disease in more than fifteen (15/36, 41%) of all included cases. The maternal COVID-19 severity of cases was as follows: asymptomatic = 9, mild = 5, or moderate = 1 [6,9,10,12,15,24,27,31,32,33]. The maternal severity of COVID-19 was reported to be severe in three cases only (3/36, 8.3%) [5,25,28]. Five studies reported to have followed measures to prevent mother-to-neonate SARS-CoV-2 transmission by immediate separation, strict isolation, no skin-to-skin contact, or breastfeeding [5,12,25,28,33], and one study reported full isolation of newborn during delivery and intensive care unit (ICU) admission [10]. Based on timing of mother-to-neonate SARS-CoV-2 transmission according to the WHO classification system, 5/36 (13.9%) were classified as confirmed intrauterine transmissions [5,9,11,12,33] and 4/36 (11.1%) were classified as possible intrauterine transmissions [14,27,30]. There were two confirmed intrapartum infections (2/36, 5.5%) [10,25], two confirmed early postpartum infections (2/36, 5.5%) [24,32], one possible intrapartum infection (1/36, 2.8%) [31], one unlikely intrapartum infection (1/36, 2.8%) [28], and one unlikely intrauterine infection (1/36, 2.8%) [29].

### 3.4. Diagnosis and Severity of SARS-CoV-2 Infection in Neonates with PPHN

Neonatal RT-PCR tests for SARS-CoV-2 were positive in six neonates only (6/36, 16.7%) [5,9,10,11,12,27], whereas a positive serology yielded neonatal SARS-CoV-2 antibodies (immunoglobulin G only) from blood specimens in eighteen cases (18/36, 50%) [6,11,13,14,15,24,31,33]. The diagnosis of neonatal SARS-CoV-2 infection was made using RT-PCR tests of the nasopharyngeal swab samples (*n* = 4) [9,10,11,12] and tracheal aspirate (*n* = 2) [11,12]. One study utilized rapid antigen tests to confirm neonatal SARS-CoV-2 positivity [5]. Only one study revealed neonatal positive results for immunoglobulin M antibodies against SARS-CoV-2 [11]. Neonatal SARS-CoV-2 positivity was confirmed in two cases using RT-PCR; however, the location of samples was not stated [5,27]. One study reported a positive SARS-CoV-2 test in a neonate on the second day of life but the method of COVID-19 testing used and the location of the sample were not mentioned [30]. Some neonates showed positive RT-PCR tests or immunoglobulin antibodies against SARS-CoV-2 at different ages: 1 day (*n* = 5) [5,9,11,12,33], 2 days (*n* = 2) [27,30], 3 days (*n* = 1) [11], 4 days (*n* = 1) [5], 5 days (*n* = 2) [10,11], 6 days (*n* = 3) [9,14,31], 7 days (*n* = 1) [11], 8 days (*n* = 1) [11], 11 days (*n* = 1) [11], 12 days (*n* = 1) [5], 13 days (*n* = 1) [12], 15 days (*n* = 1) [5], 17 days (*n* = 1) [11], and 18 days (*n* = 2) [5,24].

The most common neonatal COVID-19 symptoms were respiratory distress (12/36, 33.3%) [5,6,9,12,15,27,28,30,32,33], fever (9/36, 25%) [9,11,13,15,24,29], low O_2_ saturation (6/36, 16.7%) [5,10,25,27,29,33], shortness of breath (5/36, 13.9%) [5,13], cyanosis (5/36, 13.9%) [24,25,27,28,33], rash (5/36, 13.9%) [13,32], grunting (4/36, 11.1%) [9,10,27,28], tachypnoea (4/36, 11.1%) [9,11,27,28], retractions (3/36, 8.3%) [5,10,27], respiratory failure (3/36, 8.3%) [5,25,29], and tachycardia (3/36, 8.3%) [6,10,28]. The most common neonatal COVID-19 abnormal laboratory findings were high D-dimer (16/36, 44.4%) [6,11,13,14,15,24,28,31,32], high C-reactive protein (15/36, 41.7%) [6,9,11,14,24,27,28,29,31,33], thrombocytopenia (14/36, 38.9%) [6,10,11,24,27,28,29,32,33], high lactate dehydrogenase (13/36, 36.1%) [6,13,14,24,27,31,33], high N-terminal pro b-type natriuretic peptide (NT-proBNP) (10/36, 27.8%) [6,12,14,15,24,33], high liver function tests (10/36, 27.8%) [6,11,27,32,33], high troponin-I (8/36, 22.2%) [6,12,14,24], high ferritin (7/36, 19.4%) [9,12,14,15,24,32], high procalcitonin (6/36, 16.7%) [6,15,24,25,33], leukocytosis (6/36, 16.7%) [6,29], high interleukin-6 (5/36, 13.9%) [6,14,28], high blood urea nitrogen (5/36, 13.9%) [6,10,27], high white blood cells (4/36, 11.1%) [10,24,27,29], high prothrombin time (3/36, 8.3%) [10,25,28], lymphopenia (3/36, 8.3%) [11,28,33], and neutrophilia (3/36, 8.3%) [24,27,28]. Echocardiographic evaluation suggested PPHN in 31/36 (86.1%) [5,6,7,9,10,11,12,13,14,15,24,28,29,30,31,32,33], tricuspid regurgitation in 10/36 (27.8%) [6,9,10,31,32], dilated coronaries in 4/36 (11.1%) [13,31], coronary aneurysm 4/36 (11.1%) [6], and cardiomegaly in 2/36 (5.5%) [9,12] neonates. Chest x-rays shown cardiomegaly (19.4%) [6,31] and ground glass opacity (11.1%) [10,11,24,30]. Ultrasonography shown abnormal nonstress test (13.9%) [6].

SARS-CoV-2 infection in neonates with PPHN was severe (*n* = 16/36, 44.4%) [6,9,14,15,24,28,29,30,31,32,33] or critical (*n* = 8/36, 22.2%) [5,6,10,11,12,14,25,27], and most patients experienced ARDS (*n* = 21/36, 58.3%) [5,6,9,10,11,12,15,24,25,27,28,29,30,32,33]. Early multisystem inflammatory Syndrome in neonates (MIS-N) was reported in eighteen cases (18/36, 50%) [5,6,9,10,11,12,15,25,27,28,30,32,33] and late MIS-N in four cases (4/36, 11.1%) [6,24,29,31]. Based on the definition, a diagnosis of “most likely MIS-N” was considered in 21/36 (58.3%) cases [6,9,11,12,13,15,24,25,27,28,30,32,33], “possible MIS-N” in 8/36 (22.2%) cases [8,10,14,15,29,31], and “unlikely MIS-N” in 1/36 cases (2.8%) [5].

### 3.5. Management, Treatment Outcomes, and Relative Risk Associated with Mortality in PPHN Cases Infected with SARS-CoV-2

As expected, most prescribed pharmacotherapy agents in these PPHN cases to treat COVID-19 were antibiotics (20/36, 55.5%) [5,6,9,10,11,12,14,15,24,25,28,29,32,33], intravenous immunoglobulin (15/36, 41.7%) [9,11,12,13,14,15,27,28,31,32,33], steroids (14/36, 38.9%) [5,9,12,13,14,15,24,27,30,31], aspirin (7/36, 19.4%) [6,14,31,32], anticoagulants (3/36, 8.3%) [12,14,31], and remdesivir (2/36, 5.5%) [25,27]. Two studies reported use of prone position (*n* = 2/36, 5.5%) [25,30]. The median neonatal need of supplemental oxygen was 10 (IQR, 6 to 16), mechanical ventilation (MV) was 10.5 (IQR, 4.2 to 16.7), and inhaled nitric oxide (iNO) was 8 (IQR, 5.2 to 11.7), while the median neonatal length of stay in hospital was 20 days (IQR, 14 to 23.5). Final treatment outcomes of the neonates who had PPHN with a history of mother-to-neonate SARS-CoV-2 transmission with mortality were documented in six cases (*n* = 6/36, 16.7%) [6,10,11,12,13,14], while twenty-nine (*n* = 29/36, 80.5%) cases recovered [5,6,7,8,9,13,14,15,24,25,26,27,28,29,31,32,33], and one case was still in the ICU (*n* = 1/36, 2.8%) [30], as shown in Table 1.

Patients were stratified based on treatment outcome (survival or death). A summary of the characteristics and clinical presentation of COVID-19-infected PPHN cases that were found to be statistically significant with regards to final treatment outcome in 36 neonates who had either survived (*n* = 30) or died (*n* = 6) is shown in Table 2. Neonates in the PPHN group who had COVID-19 and died had higher rates of cardiorespiratory failure (0 vs. 33.3%) and pneumonia as a cause of PPHN (0 vs. 33.3%) compared to the neonates in the PPHN group who had COVID-19 and survived (*p* values = 0.047). Neonates with PPHN who had COVID-19 and survived received a significantly higher proportion of sildenafil (16.7% vs. 66.7%; *p* = 0.037), epinephrine (6.7% vs. 33.3%; *p* = 0.03), vasopressors (3.3 vs. 33.3%; *p* = 0.047), endothelin receptor antagonists (0 vs. 33.3%; *p* = 0.047), and antibiotics (50% vs. 83.3%; *p* = 0.048). Neonates with PPHN who had COVID-19 and died had a longer use of inhaled nitric oxide [need of iNO for 7 to <14 days: 3.3% vs. 33.3% and need of iNO for ≥14 days: 0% vs. 16.7%; *p* = 0.034]. Neonates with PPHN who had COVID-19 and died had a significantly higher percentage of SARS-CoV-2 infection with critical severity (10% vs. 83.3%) but a lower percentage of COVID-19 cases were found to be severe (50% vs. 16.7%); *p* = 0.026.

Potential determining variables associated in survival and death groups were analyzed through relative risk analysis, as shown in Table 3. The neonates who received a higher proportion of antibiotics (RR 4.14, 95% CI 0.64–6.88), neonates who had a worse severity of COVID-19 defined as critical (RR 2.84, 95% CI 0.86–9.39), neonates of the male gender (RR 2.60, 95% CI 0.30–1.17), or neonates who had a longer duration of invasive positive pressure ventilation use (RR 2.22, 95% CI 0.64–7.73) were associated with an increased relative risk for death. The relative risk of death was also high in PPHN patients infected with SARS-CoV-2 who had steroids (RR 1.87, 95% CI 0.74–4.74), NRDS as a cause of PPHN (RR 1.78, 95% CI 0.69–4.52), sildenafil to treat PPHN (RR 1.70, 95% 0.83–3.46), IVIG to treat COVID-19 (RR 1.67, 95% 0.65–4.29), and high NT-proBNP (RR 1.63, 95% CI 0.79–3.33).

## 4. Discussion

This review analyzed 36 neonates with PPHN caused by various risk factors and etiologies who also developed COVID-19. It utilized data from 21 observational studies to offer insights into the clinical progression and management outcomes. To the best of our knowledge, this is the largest review to report on the development of SARS-CoV-2 infection in neonates with PPHN. We found the main causes of PPHN in neonates who had COVID-19 were NRDS (41.7%), MSAF (16.7%), PPROM (11.1%), and HIE (5.5%). Most of the neonates diagnosed with PPHN who developed COVID-19 were ≤1-day-old (18/36, 50%), male (12/36, 33.3%), Indian ethnicity (18/36, 50%), and delivered via caesarean section (16/36, 44.4%).

COVID-19 in neonates with PPHN commonly occurred in moderate to late preterm (gestational age 32 to <37 weeks) (36.1%) or term newborns (gestational age ≥37 weeks) (25%) and neonates delivered with a normal birthweight (≥2500 g) (27.8%) or low birthweight (≥1500–2499) (25%). These findings are consistent with previous observations that shown PPHN is rare in very low birthweight neonates (birthweight < 1500 g) [34] and that the male sex is associated with an increased risk of PPHN [35,36], and usually occurs in a significant number of moderate to late preterm (gestational age 32 to <37 weeks) [37] or term (gestational age ≥ 37 weeks) neonates [35,38] or after a caesarean-section delivery [35,36]. Our results extend the findings that PPHN is infrequent in very preterm newborns (<32 weeks) [39,40] or those delivered with a low birthweight (<1500 g) [34,40]. On the contrary, PPHN was reported to be common and highly diagnosed in very preterm or low birthweight neonates [41,42,43]. The observed low rates of PPHN in very preterm newborns may be explained by the frequent use of iNO, sildenafil, and steroids [44,45,46], in addition to the low rate of refractory pulmonary hypertension to supportive cardiorespiratory care and conventional therapies [47,48,49]. A gender difference is identified in our study and more male neonates developed PPHN with COVID-19. This may be explained by the sexual dimorphism that favors structural and functional lung maturation in female neonates [50], or higher prevalence of pulmonary diseases [51,52], and increased risk for respiratory complications in male neonates [53]. However, our findings are inconsistent with previous studies that found that the prevalence of PPHN among White (Caucasian) and Black neonates was higher than other ethnicities such as Asians or Indians [54,55]. Differences in the incidence of PPHN in neonates infected with SARS-CoV-2 by ethnicity could be attributed mainly to the differences in the echocardiographic criteria used to diagnose PPHN, study design, setting, and population characteristics, or partly be explained by low socioeconomic status [56], difference in access to health care, lack of accessibility to medical treatment [57], difficulties in social distancing or being part of multigenerational or multi-family households [58], or a lack of awareness among parents when they present with symptoms of COVID-19 leading them to seek medical attention [59].

Our findings represent the most recent evidence on the co-existence of PPHN and COVID-19 along with their association with various clinical outcomes in one study. The results of our systematic review showed that PPHN in neonates who had COVID-19 was associated with increased mortality (16.7%), severity [SARS-CoV-2 infection was severe (44.4%) or critical (22.2%)], ARDS (58.3%), MIS-N (58.3%), ICU admission (58.3%), and MV usage (47.2%). It is necessary to point out that although most cases of COVID-19 in the neonatal population were asymptomatic or mild and had good prognosis [60], the overall rate of ICU admission and mortality rate we report in neonates who had PPHN and COVID-19 suggests that the risk of severe disease and mortality from SARS-CoV-2 infection is very much higher in neonates with PPHN compared to neonates without PPHN. For example, COVID-19-related ICU admission among neonates aged 11 days (IQR, 1 to 22) without PPHN who had various medical comorbidities was low (61/918, 6.6%) and the COVID-19-related death rate was reported in one neonate only from all cases with presentation suggestive of MIS-N (1/918, 0.1%) [61]. Nevertheless, our findings should be interpreted cautiously due to the small number of studies and substantial heterogeneity; thus, further research in this area is warranted.

Our findings align with some studies that found that mother-to-neonate transmission of SARS-CoV-2 is of clinical significance [62,63] and pregnant women infected with COVID-19 are at increased risk of adverse obstetrical outcomes, compared with the general population [64,65]. Among the COVID-19 positive mothers, there were cases of preeclampsia, PPROM, abortion, and death [66,67,68]. In neonates with PPHN, the SARS-CoV-2 infection outcome was associated with a relatively higher rate of ICU admission (58.3%), antibiotics use (55.5%), need of ventilatory support (47.2%), C-section delivery (44.4%), preterm birth (44.4%), neonatal respiratory distress (41.7%), and perinatal death (16.7%). Therefore, regular screening for COVID-19, early identification of infection in asymptomatic pregnant women, and safe practices to avoid acquiring an infection during early gestational ages, may be associated with more favorable pregnancy outcomes [69,70]. Mothers and their neonates should be isolated to prevent neonatal transmission, with effective protection measures during and after delivery to prevent SARS-CoV-2 transmission [71,72]. Testing for viral pathogens, including SARS-CoV-2, may be necessary for newborns showing COVID-19 symptoms, especially those with a history of exposure. Gathering illness histories from pregnant mothers, household members, and contacts is crucial to identify newborns at risk for SARS-CoV-2 infection.

In our review, neonates diagnosed with PPHN who had COVID-19 showed a positive serology for IgG SARS-CoV-2 antibodies in 50% of cases and IgM in 2.8% cases. In terms of maternal serology, some women were positive for IgG (16.7%). With the upscaling of COVID-19 vaccination in pregnancy, the possibility of maternal IgG tests being positive following vaccination needs to be considered. Only three studies reported on the vaccination status against SARS-CoV-2 for mothers who delivered PPHN patients infected with COVID-19, of which none of the mothers had received a vaccination. Therefore, it is important to document the maternal vaccination history to prevent a misdiagnosis of MIS-N [21]. Most published PPHN cases with SARS-CoV-2 reported early MIS-N diagnoses in the neonatal period. These cases are likely secondary to maternal infection during pregnancy, causing a fetal inflammatory response from transferred maternal antibodies or transplacental virus transfer [31], leading to an altered endogenous immune response [13]. Another possibility is immune dysregulation from postnatal infection [73]. To manage MIS-N caused by COVID-19 in PPHN neonates, a high proportion of patients were treated with IVIG (41.7%) and steroids (38.9%), as they have a basis in the immune-mediated hyperinflammatory pathogenesis [74,75]. A significant proportion of neonates with MIS-N received aspirin (19.4%) or anticoagulants (8.3%). Aspirin is recommended in all mild MIS-N cases to improve platelet count and prevent thrombosis, particularly with coronary artery aneurysms. Heparin is advised for moderate to severe MIS-N cases with elevated D-dimers and a low ejection fraction to prevent thrombosis [76].

We found that the mortality rate in PPHN neonates infected with COVID-19 was significantly high in patients who received sildenafil or antibiotics or neonates who had a critical severity of COVID-19. Mortality due to sildenafil in PPHN neonates infected with SARS-CoV-2 is unlikely and may be attributable to the underlying pulmonary vascular disease instead of sildenafil use [77]. Because of the overlapping clinical signs and symptoms between COVID-19 and sepsis, a high percentage of the PPHN neonates was treated with large-spectrum antibiotics (55.5%). Taking into consideration the high need for ICU admission in neonates with MIS-N (58.3%) and given that an antibiotic treatment is often guaranteed in younger infants, the length of antibiotic therapy should be judiciously evaluated in order to avoid the spread of multi-drug resistant organisms [78]. It should be noted that the difference in mortality due to PPHN based on the severity of COVID-19 might be attributed mainly to the differences in the severity of the PPHN illness and/or inclusion criteria, or the level of health care infrastructure and general care-seeking practices in low- and middle-income countries. Mortality in COVID-19 cases with PPHN included in our review might have been complicated by multi-inflammatory systemic infection in children (i.e., cytokine storm), and the patients died due to the fact of subsequent multiple organ failure induced by the viral invasion [79]. Nonetheless, PPHN is not common and has been estimated to occur in approximately two cases per one-thousand live births; however, a serious disorder of postnatal transition accounts for a significant proportion of admissions to tertiary neonatal ICUs [37]. Neonates with PPHN often require advanced cardiorespiratory support and are at significant risk of mortality and morbidities [80]. Timely diagnosis and meticulous management lead to reduced morbidities and improved clinical outcomes [81].

### Strengths and Limitations

To the best of our knowledge, this is the first systematic review conducted to review the available literature on clinical characteristics and outcomes of SARS-CoV-2 infection in neonates with PPHN. Six authors independently conducted screening, data extraction, and quality assessments to reduce selection and reviewer biases.

We recognize that our study had some limitations. First, our review is limited to published case reports of SARS-CoV-2 infection in neonates with PPHN, with most studies being retrospective. This design may introduce reporting bias due to reliance on clinical records. Second, more severe cases with worse outcomes are more likely to be published, while asymptomatic or mildly ill neonates with PPHN and COVID-19 that did not require hospitalization are underrepresented in the literature. Consequently, treatment outcomes like hospitalization, ICU admission, oxygen requirement, and death are likely overestimated. Third, a direct comparison between studies was limited, and a meta-analysis was not performed due to clinical and methodological heterogeneity, such as patients’ varying medical comorbidities and causes of PPHN. Last, we only focused on the studies that were in the English language.

## 5. Conclusions

COVID-19 in neonates with PPHN is challenging and maybe associated with increased mortality, severity, ICU admission, ARDS, MIS-N, and MV usage. The results should be interpreted with caution owing to the small number of studies and substantial heterogeneity and indicate a need for future research in this area. Due to its benefits, testing for SARS-CoV-2 should be encouraged for newborns with symptoms consistent with COVID-19, especially in neonates with a history of SARS-CoV-2 exposure. Effective protection measures should be implemented during delivery and post-delivery care as necessary.

## Figures and Tables

**Table 1:** Characteristics and clinical presentation of COVID-19-infected PPHN cases (*n* = 21 studies), 2020–2023.

Variable	All (*n* = 36) ^a^	Variable	All (*n* = 36) ^a^
Age (hours)		Maternal COVID-19 severity	
<1	10 (27.8)	Asymptomatic	9 (25)
1–3	2 (5.5)	Mild	5 (13.9)
>3	10 (27.8)	Moderate	1 (2.8)
Gender		Severe	3 (8.3)
Female	7 (19.4)	Mother-to-neonate transmission of SARS-CoV-2	
Male	12 (33.3)	Confirmed intrauterine	5 (13.9)
Ethnicity		Possible intrauterine	4 (11.1)
Indian	18 (50)	Confirmed intrapartum	2 (5.5)
Persian	2 (5.5)	Confirmed early postpartum	2 (5.5)
Bengali	1 (2.8)	Possible intrapartum	1 (2.8)
White (Caucasian)	1 (2.8)	Unlikely intrapartum	1 (2.8)
Black ^b^	1 (2.8)	Unlikely intrauterine	1 (2.8)
Hispanic	1 (2.8)	Neonatal COVID-19 symptoms	
Asian	1 (2.8)	Respiratory distress	12 (33.3)
Arab	1 (2.8)	Fever	9 (25)
Delivery mode		Low O_2_ sat	6 (16.7)
Caesarean	16 (44.4)	SOB	5 (13.9)
NSVD	2 (5.5)	Cyanosis	5 (13.9)
Induced labor	1 (2.8)	Rash	5 (13.9)
Weight (grams)		Grunting	4 (11.1)
Normal: ≥2500	10 (27.8)	Tachypnoea	4 (11.1)
Low: ≥1500–2499	9 (25)	Retractions	3 (8.3)
Very low: <1500	1 (2.8)	Respiratory failure	3 (8.3)
Extremely low: <1000	2 (5.5)	Tachycardia	3 (8.3)
Gestational age (weeks)		Neonatal abnormal laboratory findings	
Term (≥37 weeks)	9 (25)	High D-dimer	16 (44.4)
Moderate to late preterm (32 to <37 weeks)	13 (36.1)	High CRP	15 (41.7)
Very preterm (28 to <32 weeks)	3 (8.3)	Thrombocytopenia	14 (38.9)
Apgar score		High LDH	13 (36.1)
Low: 0–3	7 (19.4)	High NT-proBNP	10 (27.8)
Moderately abnormal: 4–6	11 (30.5)	High LFTs	10 (27.8)
Reassuring: 7–10	17 (47.2)	High troponin-I	8 (22.2)
Neonatal comorbidities		High ferritin	7 (19.4)
Arrhythmia	6 (16.7)	High procalcitonin	6 (16.7)
Atrial thrombi	6 (16.7)	Leukocytosis	6 (16.7)
Seizures	5 (13.9)	High IL-6	5 (13.9)
Hypotension	4 (11.1)	High BUN	5 (13.9)
ICH	4 (11.1)	High WBCs	4 (11.1)
NEC	3 (8.3)	High PTT	3 (8.3)
Sepsis	3 (8.3)	Lymphopenia	3 (8.3)
Metabolic acidosis	3 (8.3)	Neutrophilia	3 (8.3)
Decreased fetal movements	3 (8.3)	Neonatal abnormal radiological findings	
Lack of fetal movements	3 (8.3)	Echo: PPHN	31 (86.1)
Chorioamnionitis	3 (8.3)	Echo: TR	10 (27.8)
Food intolerance	3 (8.3)	CXR: Cardiomegaly	7 (19.4)
Respiratory acidosis	2 (5.5)	USG: Abnormal NST	5 (13.9)
Rash	2 (5.5)	CXR: GGO	4 (11.1)
Decreased fetal HR	2 (5.5)	Echo: Dilated coronaries	4 (11.1)
Vomiting	2 (5.5)	Echo: Coronary aneurysm	4 (11.1)
CRVS	2 (5.5)	Echo: Cardiomegaly	2 (5.5)
Shock	2 (5.5)	Neonatal COVID-19 severity	
Cardiorespiratory failure	2 (5.5)	Severe	16 (44.4)
Hydrocephalus	2 (5.5)	Critical	8 (22.2)
HIE	2 (5.5)	If neonate suffered ARDS	
Cardiogenic shock	1 (2.8)	Yes	21 (58.3)
Septic shock	1 (2.8)	No	1 (2.8)
MOD	1 (2.8)	If experienced MIS-N	
Coinfection with *Klebsiella oxytoca*	1 (2.8)	Most likely	21 (58.3)
MI	1 (2.8)	Possible	8 (22.2)
Myocarditis	1 (2.8)	Unlikely	1 (2.8)
CLD	1 (2.8)	Type of MIS-N	
PVD	1 (2.8)	Early	18 (50)
Bronchiectasis	1 (2.8)	Late	4 (11.1)
PPHN etiology		Neonatal treatment of SARS-CoV-2 infection	
NRDS	15 (41.7)	Antibiotics	20 (55.5)
MSAF	6 (16.7)	IVIG	15 (41.7)
PPROM	4 (11.1)	Steroids	14 (38.9)
HIE	2 (5.5)	Aspirin	7 (19.4)
Pneumonia	2 (5.5)	Anticoagulants	3 (8.3)
Idiopathic	1 (2.8)	Remdesivir	2 (5.5)
Initiated PPHN treatments		Prone positioning	2 (5.5)
IPPV	18 (50)	Duration on supplemental oxygen (days)	
CPAP	17 (47.2)	<7	4 (11.1)
iNO	14 (38.9)	7 to <14	9 (25)
HFV	10 (27.8)	≥14	3 (8.3)
Surfactant	9 (25)	Duration of MV (days)	
Sildenafil	9 (25)	<7	5 (13.9)
NPPV	8 (22.2)	7 to <14	8 (22.2)
Dopamine	7 (19.4)	≥14	4 (11.1)
Hydrocortisone	7 (19.4)	Duration on iNO use (days)	
Packed RBCs	6 (16.7)	<7	4 (11.1)
Milrinone	5 (13.9)	7 to <14	3 (8.3)
Inotropes	5 (13.9)	≥14	1 (2.8)
FFP	4 (11.1)	Duration of hospital stay (days)	
Diuretics	4 (11.1)	<7	0
Epinephrine	4 (11.1)	7 to <14	3 (8.3)
Dobutamine	4 (11.1)	≥14	14 (38.9)
Antihypertensives	4 (11.1)	Final treatment outcome	
Magnesium sulfate	3 (8.3)	Survived	29 (80.5)
Vasopressors	2 (5.5)	Died	6 (16.7)
Sedation	2 (5.5)	Still hospitalized	1 (2.8)
CPR	2 (5.5)		
Platelets	2 (5.5)		
ERAs	2 (5.5)		
Opioids	2 (5.5)		
Vasodilators	2 (5.5)		
Therapeutic hypothermia	2 (5.5)		
Maternal comorbidities			
Gestational DM	4 (11.1)		
PIH	3 (8.3)		
Preeclampsia	3 (8.3)		
No comorbidities	3 (8.3)		
Hypothyroidism	2 (5.5)		
Placenta previa	2 (5.5)		
Coinfection with Group B *Streptococcus*	1 (2.8)		
Coinfection with CMV	1 (2.8)		
Coinfection with *Parvovirus B19*	1 (2.8)		

Abbreviations: ARDS, acute respiratory distress syndrome; BUN, blood urea nitrogen; CLD, chronic lung disease; CMV, *Cytomegalovirus*; COVID-19, coronavirus disease 2019; CPAP, continuous positive airway pressure; CPR, cardiopulmonary resuscitation; CRP, C-reactive protein; CRVS, catecholamine-resistant vasodilatory shock; CXR, chest X-rays; DM, diabetes mellitus; ERAs, endothelin receptor antagonists; Echo, echocardiogram; FFP, fresh frozen plasma; GGO, ground-glass opacity; HFV, high frequency ventilation; HIE, hypoxic ischemic encephalopathy; HR, heart rate; ICH, intracranial hemorrhage; IL-6, interleukin 6; iNO, inhaled nitric oxide; IPPV, invasive positive pressure ventilation; LDH, lactic acid dehydrogenase; LFTs, liver function tests; MI, myocardial infarction; MIS-N, multisystem inflammatory syndrome in neonates; MOD, multiple organ dysfunction; MSAF, meconium-stained amniotic fluid; MV, mechanical ventilation; NEC, necrotizing enterocolitis; NPPV, noninvasive positive pressure ventilation; NRDS, neonatal respiratory distress syndrome; NST, nonstress test; NT-proBNP, N-terminal pro b-type natriuretic peptide; O_2_sat, oxygen saturation; PIH, pregnancy-induced hypertension; PPHN, persistent pulmonary hypertension of the newborn; PPROM, preterm premature rupture of membranes; PTT, partial thromboplastin time; PVD, pulmonary vascular disease; RBCs, red blood cells; SARS-CoV-2, severe acute respiratory syndrome coronavirus 2; SOB, shortness of breath; TR, tricuspid regurgitation; USG, ultrasonography; WBCs, white blood cells. ^a^ Data are presented as number (%). Data were calculated on patients for whom the information was available. Percentages do not total 100% owing to missing data. ^b^ Patients with black ethnicity include African-American, Black African, African, and Afro-Caribbean patients.

**Table 2:** Characteristics and clinical presentation of COVID-19-infected PPHN cases, stratified by treatment outcome (*n* = 21 studies), 2020–2023.

Variable	Findings		
	Survived (*n* = 30) ^a^	Died (*n* = 6) ^a^	*p*-Value ^b^
Neonatal comorbidities			
Cardiorespiratory failure	0	2 (33.3)	0.047 *
PPHN etiology			
Pneumonia	0	2 (33.3)	0.047 *
Initiated PPHN treatments			
Sildenafil	5 (16.7)	4 (66.7)	0.037 *
Epinephrine	2 (6.7)	2 (33.3)	0.03 *
Vasopressors	1 (3.3)	2 (33.3)	0.047 *
ERAs	0	2 (33.3)	0.047 *
Neonatal need of iNO (days)			
<7	4 (13.3)	0	0.034 *
7 to <14	1 (3.3)	2 (33.3)	
≥14	0	1 (16.7)	
Neonatal COVID-19 severity			
Severe	15 (50)	1 (16.7)	0.026 *
Critical	3 (10)	5 (83.3)	
Neonatal treatment of SARS-CoV-2 infection			
Antibiotics	15 (50)	5 (83.3)	0.048 *

Abbreviations: COVID-19, coronavirus disease 2019; ERAs, endothelin receptor antagonists; iNO, inhaled nitric oxide; PPHN, persistent pulmonary hypertension of the newborn. ^a^ Data are presented as a number (%). ^b^ Chi-square (*χ*^2^) test was used to compare between the survival and death groups. Percentages do not total 100% owing to missing data. * Represents significant differences.

**Table 3:** Relative risk for mortality among of COVID-19-infected PPHN cases (*n* = 21 studies), 2020–2023.

Variable	Survived (*n* = 30)	Died (*n* = 6)	Relative Risk	95% CIs
Gender (male)	9 (30)	3 (50)	2.60	0.30–1.17
Neonatal comorbidities (hypotension)	3 (10)	1 (16.7)	1.23	0.82–1.86
Neonatal comorbidities (sepsis)	1 (3.3)	2 (33.3)	1.28	0.85–1.93
PPHN etiology (NRDS)	13 (43.3)	2 (33.3)	1.78	0.69–4.52
PPHN treatments (sildenafil)	3 (10)	3 (50)	1.70	0.83–3.46
PPHN treatments (epinephrine)	3 (10)	1 (16.7)	1.54	0.89–2.64
PPHN treatments (HFV)	7 (23.3)	3 (50)	1.55	0.75–3.20
PPHN treatments (IPPV)	14 (46.7)	4 (66.7)	2.22	0.64–7.73
PPHN treatments (dobutamine)	2 (6.7)	2 (33.3)	1.23	0.81–1.87
PPHN treatments (surfactant)	6 (20)	3 (50)	1.24	0.70–2.20
Neonatal COVID-19 severity (critical)	3 (10)	5 (83.3)	2.84	0.86–9.39
Neonatal COVID-19 symptoms (fever)	7 (23.3)	2 (33.3)	1.24	0.70–2.20
Neonatal abnormal laboratory findings (high NT-proBNP)	7 (23.3)	3 (50)	1.63	0.79–3.33
Neonatal abnormal laboratory findings (high troponin-I)	5 (16.7)	3 (50)	1.30	0.74–2.29
Neonatal abnormal laboratory findings (high ferritin)	5 (16.7)	2 (33.3)	1.36	0.77–2.38
Neonatal abnormal radiological findings (CXR GGO)	2 (6.7)	2 (33.3)	1.28	0.85–1.92
Neonatal treatment of SARS-CoV-2 infection (IVIG)	11 (36.7)	4 (66.7)	1.67	0.65–4.29
Neonatal treatment of SARS-CoV-2 infection (steroids)	12 (40)	3 (50)	1.87	0.74–4.74
Neonatal treatment of SARS-CoV-2 infection (antibiotics)	15 (50)	5 (83.3)	4.14	0.64–6.88

Abbreviation: CIs, confidence intervals; CXR, chest X-rays; GGO, ground glass opacity; HFV, high frequency ventilation; IPPV, invasive positive pressure ventilation; NRDS, neonatal respiratory distress syndrome; NT-proBNP, N-terminal pro b-type natriuretic peptide; PPHN, persistent pulmonary hypertension of the newborn.

## Data Availability

All data generated or analyzed during this study are included in this published article.

## Supplementary Materials

The following supporting information can be downloaded at: https://www.mdpi.com/article/10.3390/children11111305/s1, Table S1: Database-specific search syntax; Table S2: Characteristics of the included studies and main outcome measures in neonates with persistent pulmonary hypertension of the newborn (PPHN) and coronavirus disease 2019 (COVID-19) (*n* = 21 studies), 2020–2023; Table S3: Clinical features in neonates with documented persistent pulmonary hypertension of the newborn (PPHN) and coronavirus disease 2019 (COVID-19) (*n* = 21 studies), 2020–2023; and Table S4: The PRISMA 2020 checklist.

## Abbreviations

hypoxic ischemic encephalopathy; MIS-N, multisystem inflammatory syndrome in neonates; MSAF, meconium-stained amniotic fluid; NEC, necrotizing enterocolitis; NRDS, neonatal respiratory distress syndrome; PPHN, persistent pulmonary hypertension of the newborn; PPROM, preterm premature rupture of membranes.
