# Supplementary material for: Clinical Characteristics and Outcomes of SARS-CoV-2 Infection in Neonates with Persistent Pulmonary Hypertension of the Newborn (PPHN): A Systematic Review
Source: Children (Basel). 2024 Oct 28;11(11):1305. doi: 10.3390/children11111305 (PMC11592555; doi:10.3390/children11111305)
Supplement: Supplementary file 1 [file children-11-01305-s001.zip › Supplementary Table S1 Database-specific search syntax.pdf]

Supplementary Table S1. Database-specific search syntax

Ovid MEDLINE(R) ALL <1946 to February 09, 2024>

1 "persistent pulmonary hypertension".mp. [mp=title, book title, abstract, original title, name of substance word, subject heading word, floating sub-heading word, keyword heading word, organism supplementary concept word, protocol supplementary concept word, rare disease supplementary concept word, unique identifier, synonyms, population supplementary concept word, anatomy supplementary concept word] 2118
2 limit 1 to english language 1975
3 "persistent fetal circulation".mp. [mp=title, book title, abstract, original title, name of substance word, subject heading word, floating sub-heading word, keyword heading word, organism supplementary concept word, protocol supplementary concept word, rare disease supplementary concept word, unique identifier, synonyms, population supplementary concept word, anatomy supplementary concept word] 1438
4 limit 3 to english language 1322
5 "pulmonary hypertension".mp. [mp=title, book title, abstract, original title, name of substance word, subject heading word, floating sub-heading word, keyword heading word, organism supplementary concept word, protocol supplementary concept word, rare disease supplementary concept word, unique identifier, synonyms, population supplementary concept word, anatomy supplementary concept word] 47058
6 limit 5 to english language 41065
7 2 or 4 or 6 41579
8 limit 7 to covid-19337

Embase <1974 to 2024 February 07>

1 exp persistent pulmonary hypertension/ 2022
2 limit 1 to english language 1961
3 "persistent fetal circulation".mp. or persistent pulmonary hypertension/ 2283
4 limit 3 to english language 2179
5 "pulmonary hypertension".mp. or exp pulmonary hypertension/ 126999
6 limit 5 to english language 114913
7 2 or 4 or 6 115065
8 limit 7 to covid-191770

CINHAL

S5 (((MH "COVID-19+") OR (MH "SARS-CoV-2")) AND (S1 OR S2 OR S3)) (26)
S4 (MH "COVID-19+") OR (MM "SARS-CoV-2") (45,845)
S3 "pulmonary hypertension" (11,189)
S2 "persistent fetal circulation" (389)
S1 "persistent pulmonary hypertension" (604)

PubMed

4 Search: ((("persistent pulmonary hypertension"[All Fields] AND "english"[Language]) OR ("persistent fetal circulation"[All Fields] AND "english"[Language]) OR ("pulmonary hypertension"[All Fields] AND "english"[Language])) AND (("COVID-19"[MeSH Terms] OR "SARS-CoV-2"[MeSH Terms] OR "pediatric multisystem inflammatory disease covid 19 related"[Supplementary Concept]) AND "english"[Language])) AND (english[Filter]) (273)
3 Search: ("pulmonary hypertension"[All Fields]) AND (english[Filter]) (51,288)
2 Search: ("persistent fetal circulation"[All Fields]) AND (english[Filter]) (1,322)
1 Search: ("persistent pulmonary hypertension"[All Fields]) AND (english[Filter]) (1,974)
