# Supplementary material for: Clinical Characteristics and Outcomes of SARS-CoV-2 Infection in Neonates with Persistent Pulmonary Hypertension of the Newborn (PPHN): A Systematic Review
Source: Children (Basel). 2024 Oct 28;11(11):1305. doi: 10.3390/children11111305 (PMC11592555; doi:10.3390/children11111305)
Supplement: Supplementary file 1 [file children-11-01305-s001.zip › Supplementary Table S2 and Supplementary Table S3 Summary of the characteristics of the included studies, main outcome measures and clinical features.pdf]

Supplementary Table S2. Characteristics of the included studies and main outcome measures in neonates with persistent pulmonary hypertension of the newborn (PPHN) and coronavirus disease 2019 (COVID-19) (n = 21 studies), 2020-2023

Author, year, study location	Study design, setting	Age (days)[a]	Male, n (%)	Ethnicity[b]	Delivery mode	Birth weight (grams)	Gestational age (weeks)	Apgar score	Neonatal comorbidities, n	PPHN aetiology	Initiated PPHN treatments, n	Maternal comorbidities, n	Maternal symptom onset for SARS-CoV-2	Maternal testing and positivity for SARS-CoV-2	Maternal COVID-19 severity
Algadeeb et al. 2020 (10), Saudi Arabia	Retrospective case report, single centre	15 minutes of age	0 (0)	1 Arab	1 C-section	≥1500-2499 g (n = 1)	1 Moderate to late preterm (32 to <37 weeks)	8 and 8 at 1 and 5 minutes	1 Metabolic acidosis<br>1 AKI<br>1 ICH<br>1 Cardiorespiratory failure<br>1 Bradycardia	1 Pneumonia<br>1 NRDS	1 Surfactant<br>1 iNO<br>1 Dobutamine<br>1 IPPV<br>1 CPAP<br>1 HFV<br>1 Hydrocortisone<br>1 Platelets<br>1 Packed RBCs<br>1 Dopamine<br>1 Epinephrine<br>1 CPR	1 PIH<br>1 Gestational DM	Within 1 day before birth	RT-PCR: NPH +ve 3 days after birth	1 Moderate
Balleda et al. 2022 (13), India	Retrospective case-series, multicentre	4 NR	4 Nptoe	4 Indians	4 NR	4 NR	4 Nptoe	4 NR	4 Seizures	4 NR	4 NPPV<br>3 IPPV<br>3 CPAP<br>3 HFV	4 NR	4 NR	4 NR	4 NR
Borkotoky et al. 2021 (24), India	Retrospective case report, single centre	4 hours of age	1 (100)	1 Indian	1 C-section	≥2500 g (n = 1)	1 Term (≥37 completed weeks' gestation)	7 and 9 at 1 and 5 minutes	1 Prolonged labour<br>1 NEC<br>1 Food intolerance<br>1 Vomiting<br>1 Rash	1 Idiopathic	1 IPPV<br>1 Sildenafil<br>1 Dopamine<br>1 Diuretics<br>1 IV fluids<br>1 HFV<br>1 CPAP	1 Gestational DM	Within 21 days before birth	IgG: Blood +ve 19 days after birth	1 Mild
Chaudhuri et al.	Prospective case-series,	4, 1, 2, 1, 1 and 3	6 NR	6 Indians	6 C-section	≥2500 g (n = 3)	3 Term (≥37 completed	At 1 minute:	6 Arrhythmia<br>6 Atrial thrombi	3 MSAF<br>2 NRDS<br>1 PPROM	6 Immunosuppressive therapies	1 IVF-ICSI pregnancy	6 NR	IgG: Blood +ve NR[§]	5 Asymptomatic

2022 (6), India	multicentre					≥1500-2499 g (n = 1)<br><1500 g (n = 1)<br><1000 g (n = 1)	weeks' gestation)<br><br>1 Moderate to late preterm (32 to <37 weeks)<br><br>2 Very preterm (28 to <32 weeks)	5, 3, 6, 3, 3 and 4<br><br>At 5 minutes: 7, 5, 8, 5, 6, and 8	2 CRVS<br>2 Decreased fetal movements<br>2 Lack of fetal movements<br>1 NEC		3 IPPV<br>2 CPAP<br>1 Epinephrine<br>1 Norepinephrine<br>1 Dopamine<br>1 Milrinone<br>1 Magnesium sulfate<br>1 Hydrocortisone<br>1 HFV<br>1 iNO	1 RhD-negative<br>1 Placenta previa		RT-PCR: (location not stated) +ve (mild COVID-19 at 29 weeks)¶	1 Mild
Easterlin et al. 2020 (25), United States	Retrospective case report, single centre	1 minute of age	0 (0)	1 NR	1 C-section	<1000 g (n = 1)	1 Very preterm (28 to <32 weeks)	0 and 2 at 1 and 5 minutes	1 No HR<br>1 Chorioamnionitis<br>1 Hypotension<br>1 ICH<br>1 TSC	1 MSAF<br>1 NRDS	1 Surfactant<br>1 HFV<br>1 iNO<br>1 Epinephrine<br>1 Hydrocortisone<br>1 Magnesium sulfate<br>1 Packed RBCs<br>1 FFP<br>1 Cryoprecipitate	1 Placenta previa<br>1 TSC<br>1 LAM<br>1 Cardiac hamartomas<br>1 Subependymal nodules<br>1 Aenoma sebaceum<br>1 Nephrectomy<br>1 Angiomyolipoma<br>1 Leiomyosarcoma<br>1 Pancreatitis	1 NR	RT-PCR: NPH +ve 6 days before admission	1 Severe

												1 Preeclampsia<br>1 Pneumothorax<br>1 Pulmonary emboli<br>1 Sepsis<br>1 Tracheostomy			
Ergon et al. 2021 (11), Turkey	Retrospective case report, single centre	1 minute of age	1 (100)	1 White (Caucasian)	1 C-section	≥1500-2499 g (n = 1)	1 Moderate to late preterm (32 to <37 weeks)	4 and 7 at 1 and 5 minutes	1 Hypotension<br>1 Sepsis	1 Pneumonia<br>1 NRDS	1 CPAP<br>1 iNO<br>1 Surfactant<br>1 HFV<br>1 Dopamine<br>1 Dobutamine<br>1 Sildenafil	1 NR	1 NR	RT-PCR: NPH +ve NR	1 Symptomatic (severity was NR)
Farmer 2021 (5), United States	Retrospective case report, single centre	1 minute of age	1 (100)	1 Black	1 C-section	≥1500-2499 g (n = 1)	1 Moderate to late preterm (32 to <37 weeks)	2 and 6 at 1 and 5 minutes	1 Hypotension<br>1 Airway oedema<br>1 Persistent hypoxia<br>1 Decreased fetal movements<br>1 Chorioamnionitis<br>1 ICH<br>1 Hydrocephalus<br>1 Respiratory acidosis<br>1 Anaemia	1 MSAF<br>1 PPROM<br>1 NRDS	1 CPR<br>1 Surfactant<br>1 CPAP<br>1 IPPV<br>1 HFV<br>1 iNO<br>1 Hydrocortisone<br>1 Dopamine<br>1 NMBAs<br>1 Opioids<br>1 Sedation<br>1 Packed RBCs<br>1 VP shunt	1 Gestational DM<br>1 Anaemia<br>1 Group B *Streptococcus* coinfection	Within 1 day after birth	RT-PCR: (location not stated) +ve 1 day at hospital admission	1 Severe

									1 Sepsis						
Gonçalves-Ferri et al. 2022 (12), Brazil	Retrospective case report, single centre	10 minutes of age	1 (100)	1 Hispanic	1 C-section	≥1500-2499 g (n = 1)	1 Moderate to late preterm (32 to <37 weeks)	8 and 9 at 1 and 5 minutes	1 Chorioamnionitis<br>1 Sepsis<br>1 Pneumothorax<br>1 Cardiorespiratory failure<br>1 Seizures<br>1 Klebsiella oxytoca coinfection<br>1 Septic shock<br>1 MI<br>1 Hypothermia	1 PPROM	1 NPPV<br>1 CPAP<br>1 Surfactant<br>1 Hydrocortisone<br>1 Sildenafil<br>1 Vasopressors<br>1 ERAs<br>1 Antihypertensives	1 Previously healthy	Within 3 days before hospital admission	RT-PCR: NPH +ve 1 day at hospital admission<br><br>WGS: P.1 (gamma) variant was detected	1 Mild
Gupta et al. 2022 (14), India	Retrospective case report, single centre	2 NR	1 (50)	2 Indians	2 NR	2 NR	2 Term (≥37 completed weeks' gestation)	2 NR	2 NR	2 NR	1 IPPV<br>2 iNO<br>1 Sildenafil<br>1 Inotropes<br>1 Vasopressors<br>1 Vasodilators<br>1 Antihypertensives<br>1 ERAs<br>1 Diuretics	2 NR	2 NR	2 NR	2 NR
Ishqeir et al. 2021 (7), Israel	Retrospective case-series,	5 NR	5 NR	5 NR	5 NR	5 NR	5 NR	5 NR	5 NR	5 NR	2 IPPV<br>1 CPAP<br>2 iNO	5 NR	5 NR	RT-PCR: (location not stated) +ve NR	5 NR

	single centre														
Jamali et al. 2023 (9), Iran	Retrospective case report, single centre	60 minutes of age	0 (0)	1 Persian	1 C-section	≥1500-2499 g (n = 1)	1 Term (≥37 completed weeks' gestation)	9 and 10 at 1 and 5 minutes	1 NEC<br>1 Transaminitis<br>1 Rash<br>1 Hydrocephalus<br>1 Cerebral palsy<br>1 ETT bleeding<br>1 Food intolerance<br>1 Vomiting	1 NRDS	1 CPAP<br>1 Surfactant<br>1 Analgesia<br>1 Inotropes<br>1 Sildenafil<br>1 Milrinone<br>1 Dopamine<br>1 Diuretics<br>1 Antihypertensives<br>1 Vitamin K<br>1 FFP<br>1 Packed RBCs	1 Previously healthy	Within 12 days before birth	RT-PCR: NPH +ve 2 days after birth	1 Mild
Joshi et al. 2022 (26), United States	Retrospective case-series, single centre	1 NR	1 (100)	1 NR	1 NR	≥2500 g (n = 1)	1 Term (≥37 completed weeks' gestation)	1 NR	1 HIE<br>1 COVID-19 exposure	1 NRDS<br>1 HIE	1 iNO<br>1 Inotropes<br>1 Vasopressin<br>1 Therapeutic hypothermia	1 NR	1 NR	1 NR	1 NR
Kalani-Moghaddam et al. 2022 (27), Iran	Retrospective case report, single centre	2 minutes of age	1 (100)	1 Persian	1 NSVD	≥2500 g (n = 1)	1 Term (≥37 completed weeks' gestation)	9 and 6 at 1 and 5 minutes	1 Respiratory acidosis	1 PPROM<br>1 MSAF	1 CPAP<br>1 NPPV<br>1 IPPV<br>1 Surfactant<br>1 Milrinone<br>1 Dopamine<br>1 Sildenafil<br>1 Dobutamine<br>1 Antihypertensives<br>1 Opioids	1 Hypothyroidism	1 NR	RT-PCR: (location not stated) +ve 3 days after birth	1 Asymptomatic

											1 Magnesium sulfate				
Malek et al. 2022 (28), Bangladesh	Retrospective case report, single centre	120 minutes of age	0 (0)	1 Bengali	1 C-section	≥1500-2499 g (n = 1)	1 Moderate to late preterm (32 to <37 weeks)	9 and 10 at 1 and 5 minutes	1 Icteric	1 NRDS	1 Routine resuscitation<br>1 CPAP<br>1 NPPV<br>1 Vasodilators<br>1 Sildenafil<br>1 Diuretics<br>1 Calcium supplements<br>1 Phototherapy<br>1 Packed RBCs<br>1 FFP<br>1 Antifungals<br>1 Calcium gluconate	1 Gestational DM<br>1 Respiratory distress<br>1 Hypothyroidism<br>1 PIH<br>1 UTI	Within 3 days before hospital admission	RT-PCR: NPH and OPS +ve 1 day at hospital admission	1 Severe
McCarty et al. 2021 (29), United States	Retrospective case report, single centre	1 minute of age	1 (100)	1 NR	1 NSVD	1 NR	1 Moderate to late preterm (32 to <37 weeks)	1 NR	1 Metabolic acidosis	1 NRDS	1 IPPV<br>1 iNO<br>1 Surfactant<br>1 Antivirals<br>1 Packed RBCs<br>1 Platelets<br>1 CPAP	1 PIH<br>1 Preeclampsia<br>1 Thrombocytopenia	Within 1 day after hospital admission	RT-PCR: NPH +ve 1 day at hospital admission	1 Symptomatic (severity was NR)
Moore and Altit 2022 (30), Canada	Retrospective case report, single centre	1 minute of age	1 (100)	1 NR	1 C-section	≥2500 g (n = 1)	1 Moderate to late preterm (32 to <37 weeks)	1 and 4 at 1 and 5 minutes	1 Decreased fetal HR<br>1 CLD<br>1 PVD<br>1 HIE<br>1 GERD<br>1 Atelectasis<br>1 Bronchiectasis	1 NRDS<br>1 HIE	1 CPAP<br>1 Therapeutic hypothermia<br>1 Sedation<br>1 iNO<br>1 Sildenafil<br>1 Milrinone	1 Previously healthy	1 NR	Type not stated: (location not stated) +ve 1 day at hospital admission<br><br>WGS: Delta variant was detected	1 NR

									1 Hyperinflation						
More et al. 2022 (15), India	Retrospective case-series, multicentre	24 hours of age (n = 1) and 4 hours of age (n = 1)	2 (100)	2 Indians	2 NR	≥2500 g (n = 2)	2 Moderate to late preterm (32 to <37 weeks)	2 NR	2 Shock<br>1 Hypotension<br>1 Transitional circulation	2 NRDS	2 IPPV<br>1 Epinephrine<br>1 Milrinone<br>1 Sildenafil	2 NR	2 NR	RT-PCR: (location not stated) +ve NR	1 Asymptomatic<br><br>1 Symptomatic (severity was NR)
Pawar et al. 2021 (31), India	Retrospective case-series, single centre	2 days of age	1 (100)	1 Indian	1 NR	≥1500-2499 g (n = 1)	1 Moderate to late preterm (32 to <37 weeks)	1 NR	1 Food intolerance<br>1 Cardiogenic shock	1 NR	1 NPPV<br>1 Inotropes	1 NR	1 NR	IgG: Blood +ve NR	1 Asymptomatic
Schoenmakers et al. 2021 (32), The Netherlands	Retrospective case report, single centre	1 minute of age	0 (0)	1 NR	1 C-section	≥1500-2499 g (n = 1)	1 Moderate to late preterm (32 to <37 weeks)	1 and 4 at 1 and 5 minutes	1 MOD<br>1 Lack of fetal movements<br>1 Hepatosplenomegaly<br>1 CAE<br>1 Decreased fetal HR<br>1 ICH	1 NRDS	1 IPPV<br>1 Surfactant<br>1 Inotropes<br>1 iNO<br>1 Hydrocortisone	1 Obesity<br>1 CMV and *ParvoB19* coinfection	Within 5 days before hospital admission	RT-PCR: NPH, OPS, blood, vaginal and urine +ve 1 day at hospital admission<br><br>RT-PCR: NPH +ve (2, 3 & 5 days) and vaginal (3 days) after birth<br><br>IHC: Placenta +ve after 6 days	1 Asymptomatic

														IgG: Blood +ve 1 day after birth	
Shaiba et al. 2021 (33), Saudi Arabia	Retrospective case report, single centre	15 minutes of age	0 (0)	1 Asian	1 Induced labour	≥2500 g (n = 1)	1 Moderate to late preterm (32 to <37 weeks)	8 and 9 at 1 and 5 minutes	1 Myocarditis<br>1 Metabolic acidosis	1 NRDS	1 IPPV<br>1 CPAP<br>1 HFV<br>1 Prostacyclin<br>1 iNO<br>1 FFP<br>1 Dobutamine<br>1 Hydrocortisone	1 Preeclampsia	Within 19 days before birth[c]	RT-PCR: NPH +ve 19 days before birth<br><br>RT-PCR: NPH +ve 10 days after birth	1 Mild
Tomar et al. 2023 (8), India	Prospective cohort, single centre	2 NR	2 Nptoe	2 Indians	2 NR	2 NR	2 NR	2 NR	2 NR	2 NR	2 NR	2 NR	2 NR	2 NR	2 NR

Abbreviations: AKI, acute kidney injury; CAE, coronary artery ectasia; CLD, chronic lung disease; CMV, *Cytomegalovirus*; COVID-19, coronavirus disease 2019; CPAP, continuous positive airway pressure; CPR, cardiopulmonary resuscitation; CRVS, catecholamine-resistant vasodilatory shock; C-section, cesarean section; DM, diabetes mellitus; ERAs, endothelin receptor antagonists; ETT, endotracheal tube; FFP, fresh frozen plasma; GERD, gastroesophageal reflux disease; HFV, high frequency ventilation; HIE, hypoxic ischemic encephalopathy; HR, heart rate; ICH, intracranial haemorrhage; ICSI, intracytoplasmic sperm injection; IgG, immunoglobulin G; IHC, immunohistochemistry; iNO, inhaled nitric oxide; IPPV, invasive positive pressure ventilation; IV, intravenous; IVF, invitro fertilization; LAM, lymphangioleiomyomatosis; MI, myocardial infarction; MOD, multiple organ dysfunction; MSAF, meconium-stained amniotic fluid; NEC, necrotizing enterocolitis; NMBAs, neuromuscular blocking agents; NPH, nasopharyngeal; NPPV, noninvasive positive pressure ventilation; Nptoe, not possible to extract; NR, not reported; NRDS, neonatal respiratory distress syndrome; NSVD, normal spontaneous vaginal delivery; OPS, oropharyngeal swab; PIH, pregnancy-induced hypertension; PPHN, persistent pulmonary hypertension of the newborn; PPROM, preterm premature rupture of membranes; PVD, pulmonary vascular disease; RBCs, red blood cells; RhD, presence of the rhesus D antigen; RT-PCR, reverse transcriptase polymerase chain reaction; SARS-CoV-2, severe acute respiratory syndrome coronavirus 2; TSC, tuberous sclerosis complex; UTI, urinary tract infection; VP, ventriculoperitoneal; WGS, whole-genomic sequencing.

[a]Age is presented for each individual patient in days (6, 31), hours (15, 24) or minutes (5, 9-12, 25, 27-30, 32, 33) in all included studies.

[b]Patients with black ethnicity include African-American, Black African, African and Afro-Caribbean patients.

[c]Repeated COVID-19 contact as mother works as a staff nurse at the same hospital (33).

[§]Maternal testing and positivity for SARS-CoV-2 by serology using blood specimens was reported in 3 cases (6).

[¶]Maternal testing and positivity for SARS-CoV-2 by RT-PCR (location was not stated) was reported in 1 case only (6).

Supplementary Table S3. Clinical features in neonates with documented persistent pulmonary hypertension of the newborn (PPHN) and coronavirus disease 2019 (COVID-19) (n = 21 studies), 2020-2023

Author, year, study location	Mother-to-neonate transmission of SARS-CoV-2	Neonatal testing for SARS-CoV-2	Neonatal COVID-19 symptoms	Neonatal abnormal laboratory findings	Neonatal abnormal radiological findings	Neonatal COVID-19 severity	If neonate suffered ARDS, n	If experienced MIS-N	MIS-N type	Neonatal treatment of SARS-CoV-2 infection, n	Neonatal need of supplemental oxygen (days)	Neonatal need of MV (days)	Neonatal need of iNO (days)	Neonatal length of stay in hospital (days)	Assessment of study risk of bias (tool used; finding); and treatment outcome
Algadeeb et al. 2020 (10), Saudi Arabia	1 Confirmed intrapartum infection	RT-PCR: NPH +ve DoL 5	1 Grunting<br>1 Retractions<br>1 Low O_2 sat<br>1 Tachycardia	1 High WBCs<br>1 Thrombocytopenia<br>1 High PTT<br>1 High creatinine<br>1 High BUN<br>1 Hyperbilirubinemia	1 CXR: GGO<br>1 Echo: PPHN<br>1 Echo: TR	1 Critical	1 Yes	1 Possible MIS-N	1 Early MIS-N	1 Antibiotics	11	11	11	11	(Modified NOS, high)<br><br>1 died
Balleda et al. 2022 (13), India	4 Not possible to determine	IgG: Blood +ve DoL NR	4 Fever<br>4 SOB<br>4 Rash	4 High D-dimer<br>4 High LDH	4 Echo: Dilated coronaries<br>4 Echo: PPHN	4 NR	4 NR	4 Most likely MIS-N	4 NR	4 IVIG<br>4 Steroids	4 NR	4 NR	4 NR	4 Nptoe	(Modified NOS, moderate)<br><br>3 survived<br>1 died[a]
Borkotoky et al. 2021 (24), India	1 Confirmed early postpartum infection	IgG: Blood +ve DoL 18	1 Fever<br>1 Cyanosis	1 High WBCs<br>1 Neutrophilia<br>1 Lymphocytosis<br>1 Thrombocytopenia<br>1 High CRP<br>1 Hypoalbuminemia<br>1 High D-dimer<br>1 High LDH<br>1 High ferritin<br>1 High CK-MB<br>1 High troponin-I<br>1 High NT-proBNP	1 CXR: GGO<br>1 Echo: PPHN	1 Severe	1 Yes	1 Most likely MIS-N	1 Late MIS-N	1 Antibiotics<br>1 Steroids	1 NR	16	1 iNO not given	34	(Modified NOS, high)<br><br>1 survived

				1 High procalcitonin											
Chaudhuri et al. 2022 (6), India	6 NR	IgG: Blood +ve DoL NR	2 Respiratory distress<br>1 Tachycardia	6 Leukocytosis<br>6 Thrombocytopenia<br>6 High CRP<br>6 High LFTs<br>5 High BUN<br>4 High D-dimer<br>4 High troponin-I<br>4 High NT-proBNP<br>3 High LDH<br>2 High procalcitonin<br>3 High IL-6	5 USG: Abnormal NST<br>2 USG: Fetal bradycardia<br>1 USG: IUGR<br>1 USG: PE<br>6 CXR: Cardiomegaly<br>6 Echo: PPHN<br>6 Echo: TR<br>4 Echo: Coronary aneurysm	5 Severe<br><br>1 Critical	6 Yes	6 Most likely MIS-N	5 Early MIS-N<br><br>AND<br><br>1 Late MIS-N	6 Antibiotics<br>4 Aspirin	>7 days (n = 5)	>7 days (n = 5)	6 NR	12, 14, 20, 19, 20 and 12	(Modified NOS, moderate)<br><br>5 survived<br>1 died[b]
Easterlin et al. 2020 (25), United States	1 Confirmed intrapartum infection	RT-PCR: TA -ve DoL 1<br><br>RT-PCR: NPH -ve DoL 1 and 10	1 Low O_2 sat<br>1 Apnoea<br>1 Cyanosis<br>1 Respiratory failure	1 High PT<br>1 High INR<br>1 High PTT<br>1 High fibrinogen<br>1 High procalcitonin	1 CXR: NRDS (COVID-19)	1 Critical	1 Yes	1 Most likely MIS-N	1 Early MIS-N	1 HCQ<br>1 Antibiotics<br>1 Remdesivir<br>1 Tocilizumab<br>1 CP<br>1 Prone positioning	10	10	10	1 NR	(Modified NOS, moderate)<br><br>1 survived

Ergon et al. 2021 (11), Turkey	1 Confirmed intrauterine infection	RT-PCR: NPH +ve DoL 1, 3, 8, 11 and 17<br><br>RT-PCR: TA +ve DoL 7<br><br>IgG: Blood +ve DoL 5<br><br>IgM: Blood +ve DoL 5	1 Fever<br>1 Tachypnoea	1 Thrombocytopenia<br>1 Lymphopenia<br>1 High CRP<br>1 High LFTs<br>1 High D-dimer	1 CXR: GGO<br>1 CXR: Pneumonia (COVID-19)<br>1 Echo: PPHN	1 Critical	1 Yes	1 Most likely MIS-N	1 Early MIS-N	1 Antibiotics<br>1 IVIG<br>1 LPV/RTV	16	17	12	17	(Modified NOS, high)<br><br>1 died
Farmer 2021 (5), United States	1 Confirmed intrauterine infection	RATs: (location not stated) +ve DoL 1, 4, 12, 15 and 18<br><br>RT-PCR: (location not stated)	1 Retractions<br>1 SOB<br>1 Hypoxia<br>1 Hypercarbia<br>1 Respiratory distress<br>1 Low O_2 sat<br>1 Respiratory failure	1 Low haematocrit	1 CXR: Complete white-out<br>1 CXR: Hazy opacities<br>1 CXR: NRDS (COVID-19)<br>1 Echo: PPHN	1 Critical	1 Yes	1 Unlikely MIS-N	1 Early MIS-N	1 Antibiotics<br>1 Steroids	19	11	5	20	(Modified NOS, moderate)<br><br>1 survived

		+ve DoL 4													
Gonçalves-Ferri et al. 2022 (12), Brazil	1 Confirmed intrauterine infection	RT-PCR: NPH +ve DoL 1<br><br>RT-PCR: TA +ve DoL 13<br><br>WGS: P.1 (gamma) variant was detected	1 Respiratory distress<br>1 Hypoxia	1 High ferritin<br>1 High troponin-I<br>1 High NT-proBNP	1 CXR: IPIs<br>1 CXR: Coalescent opacities<br>1 Echo: PPHN<br>1 Echo: Cardiomegaly	1 Critical	1 Yes	1 Most likely MIS-N	1 Early MIS-N	1 Antibiotics<br>1 IVIG<br>1 Anticoagulants<br>1 Steroids	4	74	53	74	(Modified NOS, high)<br><br>1 died
Gupta et al. 2022 (14), India	2 Possible intrauterine infection	IgG: Blood +ve DoL NR<br><br>IgG: Blood +ve DoL 6	2 NR	1 High D-dimer<br>2 High troponin-I<br>2 High ferritin<br>2 High LDH<br>1 High NT-proBNP<br>1 High CPK<br>1 High CRP<br>1 High IL-6	2 Echo: PPHN	1 Severe<br><br>1 Critical	2 NR	2 Possible MIS-N	2 NR	1 Antibiotics<br>2 IVIG<br>1 Steroids<br>1 Anticoagulants<br>1 Aspirin	2 NR	2 NR	2 NR	2 NR	(Modified NOS, moderate)<br><br>1 survived<br>1 died
Ishqeir et al. 2021 (7), Israel	5 NR	5 NR	5 NR	5 NR	5 Echo: PPHN	5 NR	5 NR	5 NR	5 NR	5 NR	5 NR	5 NR	5 NR	5 NR	(Modified NOS, moderate)<br><br>5 survived
Jamali et al. 2023 (9), Iran	1 Confirmed intrauterine infection	RT-PCR: NPH	1 Fever<br>1 Grunting	1 High CRP<br>1 High ferritin	1 Echo: PPHN	1 Severe	1 Yes	1 Most likely MIS-N	1 Early MIS-N	1 Antibiotics	1 NR	22	1 iNO not given	29	(Modified NOS, high)

		+ve DoL 1 and 6	1 Respiratory distress<br>1 Tachypnoea		1 Echo: Cardiomegaly<br>1 Echo: Opacification of lungs<br>1 Echo: Valvular regurgitation<br>1 Echo: MR<br>1 Echo: TR					1 IVIG<br>1 Steroids					1 survived
Joshi et al. 2022 (26), United States	1 NR	1 NR	1 NR	1 NR	1 NR	1 NR	1 NR	1 NR	1 NR	1 NR	1 NR	1 NR	1 NR	1 NR	(Modified NOS, moderate)<br><br>1 survived
Kalani-Moghaddam et al. 2022 (27), Iran	1 Possible intrauterine infection	RT-PCR: (location not reported) +ve DoL 2	1 Respiratory distress<br>1 Cyanosis<br>1 Low O_2 sat<br>1 Tachypnoea<br>1 Retractions<br>1 Grunting	1 High WBCs<br>1 Neutrophilia<br>1 Lymphocytosis<br>1 Thrombocytopenia<br>1 High BUN<br>1 High LFTs<br>1 High LDH<br>1 High CRP	1 CXR: PGIs	1 Critical	1 Yes	1 Most likely MIS-N	1 Early MIS-N	1 Remdesivir<br>1 IVIG<br>1 Steroids	13	4	1 iNO not given	20	(Modified NOS, high)<br><br>1 survived
Malek et al. 2022 (28), Bangladesh	1 Unlikely intrapartum infection	RT-PCR: NPH -ve DoL 1 and 18	1 Tachypnoea<br>1 Tachycardia<br>1 Chest indrawing	1 Neutrophilia<br>1 Lymphopenia<br>1 High CRP<br>1 Thrombocytopenia<br>1 Hyperbilirubinemia	1 CXR: Normal<br>1 Echo: VSD<br>1 Echo: ASD	1 Severe	1 Yes	1 Most likely MIS-N	1 Early MIS-N	1 Antibiotics<br>1 IVIG	20	1 No MV	1 iNO not given	24	(Modified NOS, high)<br><br>1 survived

			1 Grunting<br>1 Cyanosis<br>1 Respiratory distress	1 High D-dimer<br>1 High PT<br>1 High PTT<br>1 High INR<br>1 High IL-6<br>1 Hypoalbuminemia<br>1 Low Hb	1 Echo: PPHN										
McCarty et al. 2021 (29), United States	1 Unlikely intrauterine infection	RT-PCR: Nasal -ve DoL 1 and 2	1 Fever<br>1 Low O_2 sat<br>1 Respiratory failure	1 Thrombocytopenia<br>1 Leukocytosis<br>1 High WBCs<br>1 High CRP	1 CXR: Granular opacities<br>1 Echo: PPHN	1 Severe	1 Yes	1 Possible MIS-N	1 Late MIS-N	1 Antibiotics	9	5	6	22	(Modified NOS, high)<br><br>1 survived
Moore and Altit 2022 (30), Canada	1 Possible intrauterine infection	Type not stated: (location not stated) +ve DoL 2<br><br>WGS: Delta variant was detected	1 Cough<br>1 Respiratory distress	1 NR	1 CXR: GGO<br>1 CXR: Fibrotic changes<br>1 CT: DLI<br>1 Echo: PPHN	1 Severe	1 Yes	1 Most likely MIS-N	1 Early MIS-N	1 Prone positioning<br>1 Steroids<br>1 Gabapentin	6	4	1 iNO not given	>5 months	(Modified NOS, high)<br><br>1 still ongoing
More et al. 2022 (15), India	2 Not possible to determine	IgG: Blood +ve DoL NR	2 Respiratory distress<br>1 Lethargy<br>1 Fever	1 High procalcitonin<br>2 High D-dimer<br>1 High ferritin<br>2 High NT-proBNP	2 Echo: PPHN	2 Severe	2 Yes	1 Most likely MIS-N<br><br>AND	2 Early MIS-N	2 Antibiotics<br>2 Steroids<br>1 IVIG	2 NR	2 NR	2 iNO not given	2 NR	(Modified NOS, high)<br><br>2 survived

								1 Possible MIS-N							
Pawar et al. 2021 ([31](#)), India	1 Possible intrapartum infection	IgG: Blood +ve DoL 6	1 NR	1 High D-dimer<br>1 High LDH<br>1 High CRP	1 CXR: Cardiomegaly<br>1 Echo: Dilated coronaries<br>1 Echo: TR<br>1 Echo: ASD<br>1 Echo: MR<br>1 Echo: PPHN	1 Severe	1 No	1 Possible MIS-N	1 Late MIS-N	1 Steroids<br>1 IVIG<br>1 Aspirin<br>1 Anticoagulants	1 NR	1 No MV	1 iNO not given	14	(Modified NOS, high)<br><br>1 survived
Schoenmakers et al. 2021 ([32](#)), The Netherlands	1 Confirmed early postpartum infection	RT-PCR: All NPH, CB, faeces, urine, blood and sputum samples were -ve over a period of 9 days<br><br>IgG: CB -ve DoL	1 Respiratory distress<br>1 Rash	1 High LFTs<br>1 High cardiac enzyme<br>1 High creatinine<br>1 Thrombocytopenia<br>1 Leukocytopenia<br>1 High D-dimer<br>1 High ferritin	1 CXR: Bilateral opacities<br>1 Echo: PPHN<br>1 Echo: TR	1 Severe	1 Yes	1 Most likely MIS-N	1 Early MIS-N	1 Antibiotics<br>1 IVIG<br>1 Aspirin	6	6	6	1 NR	(Modified NOS, high)<br><br>1 survived

		1, 3 and 21													
Shaiba et al. 2021 (33), Saudi Arabia	1 Confirmed intrauterine infection	RT-PCR: NPH -ve DoL 1, 3, 4, 5, 6, 7, 8 and 12<br><br>IgG: Blood +ve DoL 1	1 Cyanosis<br>1 Respiratory distress<br>1 Low O_2 sat	1 Lymphopenia<br>1 Thrombocytopenia<br>1 Low Hb<br>1 High LFTs<br>1 High LDH<br>1 High CK-MB<br>1 High NT-proBNP<br>1 High procalcitonin<br>1 High CRP	1 Echo: PPHN	1 Severe	1 Yes	1 Most likely MIS-N	1 Early MIS-N	1 Antibiotics<br>1 IVIG	6	4	3	1 NR	(Modified NOS, high)<br><br>1 survived
Tomar et al. 2023 (8), India	2 Not possible to determine	RT-PCR: (location not stated) -ve DoL NR	2 Nptoe	2 Nptoe	2 Nptoe	2 Nptoe	2 Nptoe	2 Possible MIS-N	2 NR	2 Immunomodulators	2 NR	2 NR	2 NR	2 NR	(NOS, 6)<br><br>2 survived

Abbreviations: ARDS, acute respiratory distress syndrome; ASD, atrial septal defect; BUN, blood urea nitrogen; CB, cord blood; CK-MB, creatine kinase myocardial band; COVID-19, coronavirus disease 2019; CP, convalescent plasma; CPK, creatinine phosphokinase; CRP, C-reactive protein; CT, computed tomography; CXR, chest x-rays; DLI, diffuse lung injury; DoL, day(s) of life; echo, echocardiogram; GGO, ground-glass opacity; Hb, haemoglobin; HCQ, hydroxychloroquine; IgG, immunoglobulin G; IgM, immunoglobulin M; IL-6, interleukin 6; iNO, inhaled nitric oxide; INR, international normalized ratio; IPIs, interstitial pulmonary infiltrates; IUGR, uterine growth restriction; IVIG, intravenous immunoglobulin; LDH, lactic acid dehydrogenase; LFTs, liver function tests; LPV/RTV, lopinavir-ritonavir; MIS-N, multisystem inflammatory syndrome in neonates; MR, mitral regurgitation; MV, mechanical ventilation; NOS, Newcastle–Ottawa Scale; NPH, nasopharyngeal; Nptoe, not possible to extract; NR, not reported; NST, nonstress test; NT-proBNP, N-terminal pro b-type natriuretic peptide; O_2sat, oxygen saturation; PE, pulmonary embolism; PGIs, patchy granular infiltrates; PPHN, persistent pulmonary hypertension of the newborn; PT, prothrombin time; PTT, partial thromboplastin time; RATs, rapid antigen tests; RT-PCR, reverse transcriptase polymerase chain reaction; SARS-CoV-2, severe acute respiratory syndrome coronavirus 2; SOB, shortness of breath; TA, tracheal aspirate; TR, tricuspid regurgitation; USG, ultrasonography; VSD, ventricular septal defect; WBCs, white blood cells; WGS, whole-genomic sequencing.

[a]Patient had multisystem illness (respiratory, cardiovascular, central nervous system and renal involvement) (13).

[b]Patient was born extreme preterm and expired from necrotizing enterocolitis on the 20th day of life (6).
